# Synthesis of mixed-sequence oligonucleotides on mesoporous silicon: chemical strategies and material stability

**DOI:** 10.1186/1556-276X-9-317

**Published:** 2014-06-25

**Authors:** Monica Terracciano, Ilaria Rea, Luca De Stefano, Ivo Rendina, Giorgia Oliviero, Fabrizia Nici, Stefano D'Errico, Gennaro Piccialli, Nicola Borbone

**Affiliations:** 1 Institute for Microelectronics and Microsystems, National Council of Research, Naples 80131, Italy; 2 Department of Pharmacy, University of Naples Federico II, Naples 80138, Italy

**Keywords:** Mesoporous silicon functionalization, DNA synthesis, Deprotection conditions, Surface stability

## Abstract

Rapid screening tests in medical diagnostic and environmental analysis are often based on oligonucleotide biochips. In this paper, we studied the stability of functionalized mesoporous silicon supports in the solid-phase synthesis of oligonucleotides, exploiting several chemical procedures. A 19-mer mixed sequence has been successfully synthesized on aminosilane-modified porous silicon photonic structures. The process and the materials have been characterized by optical reflectivity, atomic force microscopy and high-performance liquid chromatography.

## Background

DNA chip technology has greatly evolved over the last decade, moving from pure genomics towards a number of biotechnology applications such as human disease diagnostics [1], environmental monitoring and food control [2,3]. DNA chips can be classified as a special class of biosensors since they are realized by immobilization of single-stranded oligonucleotides (ONs), the bioprobe, on a transducer surface. Any molecular interaction between the bioprobe and its ligands, such as hybridization to the complementary DNA sequence or protein binding, is then transduced into an analytical signal by an electrochemical-, optical- or surface plasmon resonance-based or electrical device, depending on the specific technology used. Porous silicon (PSi) is by far one of the most popular transducer materials due to its peculiar physical and chemical properties [4]. PSi is fabricated by electrochemical etching of crystalline silicon in aqueous hydrofluoric acid. Depending on etch time, current density and acid concentration, several porous morphologies can be obtained, from micropores (average pore size <5 nm) to macropores (average pore size >50 nm) [5]. The resulting sponge-like matrix possesses a very large specific surface area (up to 300 m^2^/cm^3^): gases and liquids can easily get into pores, thus changing the optical, chemical and electrical properties of PSi [6]. Even if electrochemical etching induces silicon dissolution, the resulting PSi surface is smooth enough to get very good quality optical devices, also in the case of multilayered structures [7]. Periodic, or quasi-periodic, alternation of high- and low-porosity layers is used for fabrication of Bragg reflectors, microcavities and Thue-Morse sequences: all these photonic devices exhibit resonance wavelengths that can be used as monitoring peak in quantifying biomolecular interaction from the optical point of view [8-10]. The PSi surface can be properly passivated and functionalized in order to covalently bind biological molecules such as single- or double-stranded DNA, proteins, enzymes, antibodies, aptamers and so on, which act as bioprobes. There are many routes to achieve surface functionalization which are based on proper chemical or biological processes: the PSi surface can be activated by specific chemical groups, namely -SH, -NH_2_ or -COOH, that could form very stable bonds, such as sulphide or peptide bond, with the biological molecule considered [11]. For some biomolecules that are usually synthesized *ex situ* and then coupled on the PSi surface, there is also the possibility of directly growing the molecules using PSi as support in the so-called solid-phase synthesis [12].

In this article, we describe the fabrication and the characterization of a PSi-based DNA chip for biochemical optical sensing through *in situ* mixed-sequence ON growth. Since the chemistry used for the solid-phase synthesis of ON can be quite aggressive against the PSi solid support, the chemical stability of PSi supports is a key issue that must be checked and satisfied for each considered substrate. In particular, it is well known that PSi suffers upon exposure to alkaline solutions (commonly used for the deprotection of nucleobases) that can easily corrode the silicon skeleton, so a trade-off between PSi surface passivation and suitable solid-phase synthesis chemistry must be found. We focused our studies on silanization of PSi by using two different siloxanes and also on the exploitation of different chemical approaches for the ON deprotection in order to preserve the stability of PSi during all phases of synthesis and sensing.

## Methods

### Mesoporous silicon microcavity fabrication

PSi microcavities constituted by a *λ*/2 layer (optical thickness) sandwiched between two 9.5-period Bragg reflectors (BRs) were obtained alternating low (L) and high (H) refractive index layers whose thicknesses satisfy the Bragg relationship *n*_H_*d*_H_ + *n*_L_*d*_L_ = *mλ*_B_/2, where *m* is an integer and *λ*_B_ is the Bragg wavelength. The microcavities were prepared by electrochemical etching of highly doped p^+^ crystalline silicon (0.001-Ω cm resistivity, <100 > -oriented, 500 μm thick) in HF solution (HF:ethanol 1:1) in the dark at room temperature (RT). Before the anodization process, the silicon substrate was immersed in HF solution for 2 min to remove the native oxide layer. Since the PSi fabrication process is self-stopping, it is possible to obtain adjacent layers with different porosities by changing the current density during the electrochemical etching [4]. A current density of 200 mA/cm^2^ for 1.2 s was applied to obtain low refractive index layers (*n*_L_ = 1.542; *d*_L_ = 125 nm) while a current density of 100 mA/cm^2^ was applied for 1.4 s for high refractive index layers (*n*_H_ = 1.784; *d*_H_ = 108 nm). After the electrochemical process, the pore dimension was increased to favour the infiltration of biological matter by rinsing the fresh-made PSi microcavities in a KOH ethanol solution (1.5 mM) for 15 min [5]. The structures were then thermally oxidized against uncontrolled environmental aging and corrosion in alkaline solutions. The thermal oxidation has been performed in pure O_2_ by a two-step process: pre-oxidation at 400°C for 30 min followed by oxidation at 900°C for 15 min.

### Silane surface modifications

Eight oxidized PSi microcavities (PSi-M_a-h_) were immersed in piranha solution (H_2_O_2_:H_2_SO_4_ 1:4) at RT for 30 min to generate Si-OH groups on the PSi surface. After that, the samples were extensively washed in Milli-Q® water flow (Millipore, Billerica, MA, USA) and dried with nitrogen gas. Structures were then silanized by immersion in different 5% aminosilane solutions, (3-aminopropyl)triethoxysilane (APTES) or (3-aminopropyl)-dimethyl-ethoxysilane (APDMES), in dry toluene for 30 min at RT. Samples PSi-M_a,c,e,g_ were silanized by APTES and samples PSi-M_b,d,f,h_ by APDMES. The reaction conditions were optimized on a crystalline silicon-varying solvent for silane dissolution and incubation time [12]. The PSi-silanized samples were rinsed three times in the solvent used for the process so as to remove the ungrafted silanes. The last step of silanization is curing at 100°C for 10 min.

### Oligonucleotide synthesis

**Figure 1:**
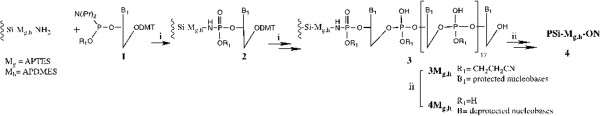
**Synthetic procedure for solid-phase synthesis of aminosilane-modified mesoporous silicon.** (i) Standard procedure for automated ON synthesis. (ii) NH_3_/MeOH dry.

Chemicals and solvents were purchased from Sigma-Aldrich (St. Louis, MO, USA). Reagents and phosphoramidites for DNA synthesis were purchased from Glen Research (Sterling, VA, USA). Solid-phase ON syntheses were performed on a PerSeptive Biosystem Expedite 8909 DNA automated synthesizer (Framingham, MA, USA). The 19-mer mixed-sequence oligonucleotide 5′-GATTGATGTGGTTGATTTT-3′ was assembled on two different aminosilane-modified microcavities, following phosphoramidite chemistry by 19 growing cycles [13]. PSi structures, PSi-M_g,h_-NH_2_ (M_g_ = APTES, M_h_ = APDMES), were introduced in a suitable column reactor to be used in the automated synthesizer; the syntheses were performed according to the scheme reported in Figure 1. In all cases, the first reaction step involved the attachment of the 3′-ending nucleobase to the amino group of PSi-bound APTES or APDMES. This step required the activation of the protected phosphoramidite dissolved in dry acetonitrile via protonation by weakly acidic tetrazole (0.45 M in acetonitrile). Once the first nucleobase was installed on the solid support, the ON growth was obtained by repeating the following sequential steps of the automated ON synthesis:

● Coupling: reaction of the protected phosphoramidite dissolved in dry acetonitrile and activated via protonation by weakly acidic tetrazole (0.45 M in acetonitrile) with the 5′-OH ON terminal group.

● Oxidation: oxidation of the unstable phosphite triester linkage to the more stable phosphotriester by a standard oxidizing solution of iodine in pyridine/acetonitrile.

● Capping: acylation of the unreacted 5′-OH ON terminal groups by acetic anhydride in pyridine and tetrahydrofuran to minimize deletion products and simplify the purification process.

● Detritylation: removal of the 5′-dimethoxytrityl (DMT) protecting group from the support-bound 5′-terminal nucleotide with the deblocking solution of trichloroacetic acid in dichloromethane (3% *w*/*w*).

The amount of DMT cation released by acid treatment was used as a direct measure of the efficiency of the ongoing synthesis. The release of the protecting group generates a bright red-orange colour solution in which the quantity of the DMT cation can be measured online by UV-vis spectroscopy at 495 nm (*ϵ* = 71,700 M^−1^ cm^−1^). At the end of each growing cycle, the support was thoroughly washed with acetonitrile before the beginning of the successive cycle.

### Deprotection strategies

The devices PSi-M_a,b_-NH_2_ (M_a_ = APTES, M_b_ = APDMES) were left in contact with 33% aqueous ammonia at 55°C for different times to investigate the effect of standard ON deprotection condition (55°C for 17 h) on the PSi matrix [14]. Two additional aminosilane-modified devices, PSi-M_c,d_-NH_2_, (M_c_ = APTES, M_d_ = APDMES) were incubated in anhydrous K_2_CO_3_ (0.05 M)/dry methanol solution at 55°C for different times to investigate the ‘ultra-mild’ ON deprotection condition (55°C for 2 h) [14]. Finally, the exposure to dry ammonia solution (NH_3_/MeOH dry) was also explored as an alternative deprotection strategy [15]. To this aim, the aminosilane-modified samples PSi-M_e,f_-NH_2_ (M_e_ = APTES, M_f_ = APDMES) were exposed to dry ammonia overnight at RT. The dry ammonia was generated by dissolving NaOH pellets in a sidearm flask containing aqueous ammonia; the generated gas was passed through a KOH drying tube and bubbled into a flask equipped with a rubber septum and containing anhydrous MeOH at 0°C.

The explored deprotection strategies carried out on aminosilane-modified PSi microcavities are summarized in Table 1.

**Table 1:** Deprotection strategies

**Deprotection strategy**	**Exposition time**	**Sample**
NH_3_(l) @55°C	30 min; 1 h; 2 h	PSi-M_a-NH2_
		PSi-M_b-NH2_
K_2_CO_3_/MeOH @55°C	30 min; 1 h; 2 h; 5 h; 8 h; overnight	PSi-M_c-NH2_
		PSi-M_d-NH2_
NH_3_(g) @RT	Overnight	PSi-M_e-NH2_
		PSi-M_f-NH2_
		PSi-M_g-NH2-oligo_
		PSi-M_h-NH2-oligo_

### Atomic force microscopy

A XE-100 AFM (Park Systems, Suwon, South Korea) was used to study sample morphology. Surface imaging was obtained in non-contact mode using silicon/aluminium-coated cantilevers (PPP-NCHR 10 M, Park Systems, Suwon, South Korea) 125 mm long with a resonance frequency of 200 to 400 kHz and nominal force constant of 42 N/m. The scan frequency was typically 1 Hz per line. The scan area in surface analysis was 1 μm × 1 μm.

### Spectroscopic reflectometry

Reflectivity spectra of PSi optical structures were obtained by a simple experimental setup: a white light was sent on PSi samples through a Y optical fibre (Avantes, Apeldoorn, The Netherlands). The same fibre was used to guide the output signal to an optical spectrum analyser (Ando AQ6315A, Tokyo, Japan). The spectra were acquired at normal incidence over the range 600 to 1,200 nm with a resolution of 5 nm. The reflectivity spectra shown in the graphs are the average of three measurements for each sample.

### High-performance liquid chromatography

The purification and control of the synthesized ONs was carried out using a Jasco PU2089 PLUS HPLC system (Easton, MD, USA) equipped with an anion exchange column (1000-8/46, 4.4 × 50 mm, 5 μm, Macherey-Nagel, Düren, Germany) using a linear gradient from 0% to 100% B in 30 min, flow rate = 1 mL/min and detection at 260 nm (buffer A: 20 mM NaH_2_PO_4_ aq. solution, pH 7.0, containing 20% (*v*/*v*) CH_3_CN; buffer B: 20 mM NaH_2_PO_4_ aq. solution, pH 7.0, containing 1 M NaCl and 20% (*v*/*v*) CH_3_CN).

## Results and discussion

In our previous work [16], we investigated the passivation ability of oxidized PSi multilayered structures by two aminosilane compounds (APTES and APDMES) used for the *in situ* synthesis of a 13-mer polythymine ON strand. We successfully demonstrated that even using the less aggressive carbonate/methanol solution as the ON deprotection system, hybridization with the complementary ON target took place, thus confirming that ONs can be synthesized and deprotected on the PSi surface. However, the synthesis of mixed-sequence ONs using the carbonate/methanol solution in the final ON deprotection step would require the use of highly expensive ultra-mild nucleobase-protected phosphoramidites characterized by having non-standard very labile protecting groups. In the present paper, we describe the results of alternative PSi-friendly ON deprotection conditions during the *in situ* synthesis of mixed-sequence ONs on PSi supports by using standard phosphoramidite nucleoside monomers, without using ultra-mild reagents.

**Figure 2:**
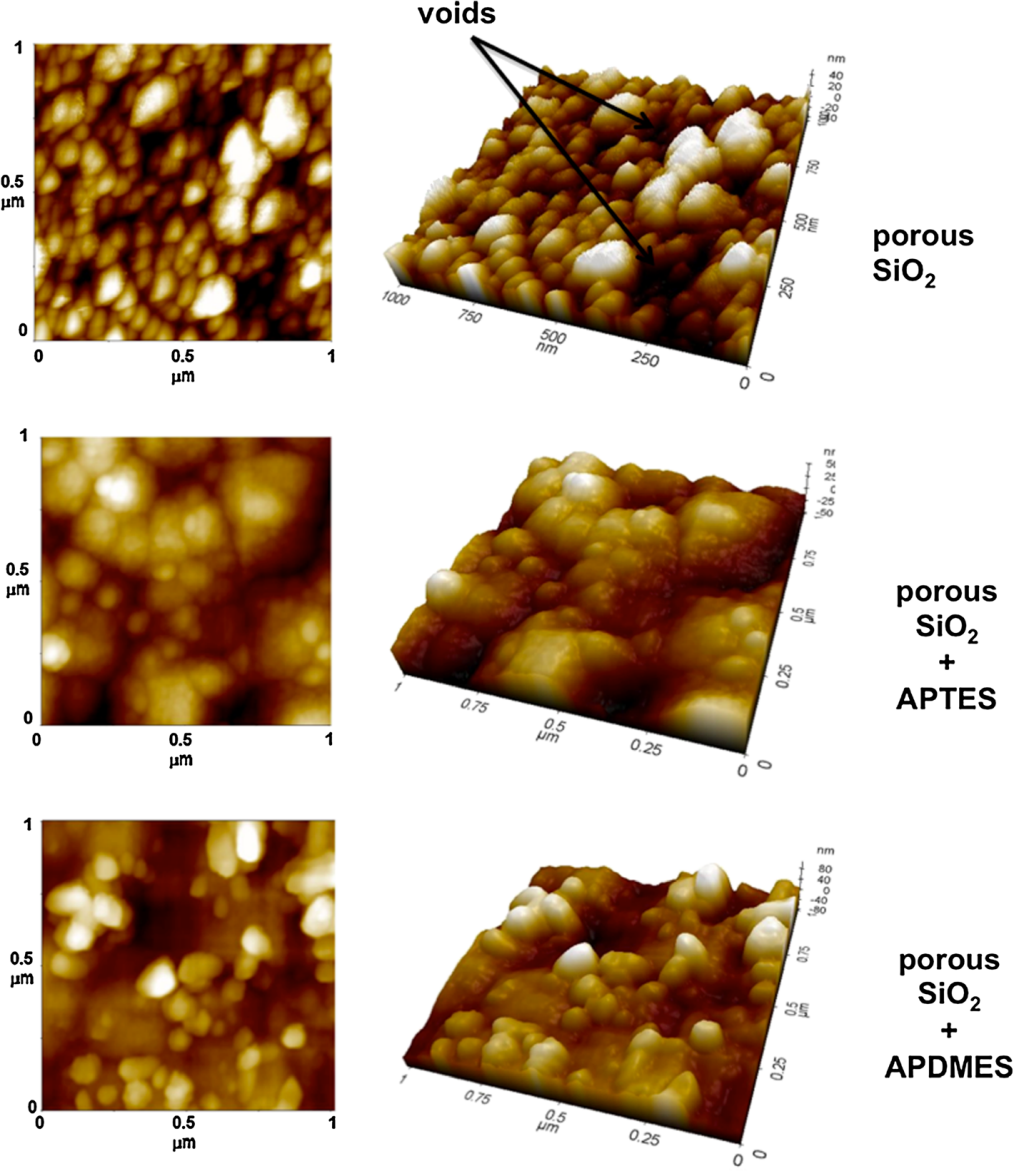
AFM images of bare oxidized PSi and aminosilane-modified oxidized PSi surfaces.

Measurement of optical spectra by spectroscopic reflectometry is very useful since both the position of resonance wavelength and the shape of lateral fringes give quantitative information about PSi corrosion or stability: the peak wavelengths of each PSi-M_a-h_ microcavity before and after silanization are reported in Table 2. Both APTES and APDMES covered the pore walls by a thin film: the aminosilane layer substitutes air in the pores and increases the average refractive index of PSi layers, resulting in a shift of reflectivity spectra towards greater wavelengths. The thicknesses of the APTES and APDMES layers coating the pore walls were estimated from red shifts: in the first case, we observed a 22 nm red shift, corresponding to a silane layer of 0.7 nm; in the second, the red shift was about 10 nm, corresponding to a silane layer of 0.2 nm [16]. These numbers are consistent with the different behaviours of the polymers: APTES generally cross-links after curing, producing a compact and thicker sheet of silane, whereas APDMES does not polymerize. A direct evidence of the slightly distinct morphologies of aminosilane-modified surfaces was given by atomic force microscopy (AFM). The AFM images of bare oxidized PSi and APTES- and APDMES-modified porous PSi surfaces are reported in Figure 2. The AFM image of porous SiO_2_ reveals a sponge-like structure characterized by hillocks and voids randomly distributed on the whole surface; pore size can be estimated to be on the order of 20 nm. After APTES grafting (porous SiO_2_ + APTES), most voids disappear due to partial pore cloaking by the silane layer coating the pore walls. Quite the same result is obtained in the case of APDMES modification (porous SiO_2_ + APDMES): even if APDMES forms a thinner layer, voids in the porous matrix are strongly reduced. Further investigations about the effect of this steric hindrance on oligonucleotide synthesis are also required.

**Table 2:** Peak shift of devices after surface modification by APTES or APDMES

**Sample**	**Pre-silanization**	**Post-silanization**	**Peak shift (nm)**
	**Peak wavelength (nm) Er±**	**Peak wavelength (nm) Er±**	
PSi-M_a_	631.3 ± 0.3	653.3 ± 0.1	22.2
PSi-M_b_	640.1 ± 0.1	651.0 ± 0.2	11
PSi-M_c_	635.7 ± 0.5	656.9 ± 0.4	21.2
PSi-M_d_	628.4 ± 0.6	640.7 ± 0.3	12.3
PSi-M_e_	708.2 ± 0.2	730.3 ± 0.6	22.3
PSi-M_f_	714.7 ± 0.1	722.3 ± 0.4	8
PSi-M_g_	706.5 ± 0.3	727.8 ± 0.1	21.3
PSi-M_h_	665.6 ± 0.4	673.7 ± 0.2	8.1

**Figure 3:**
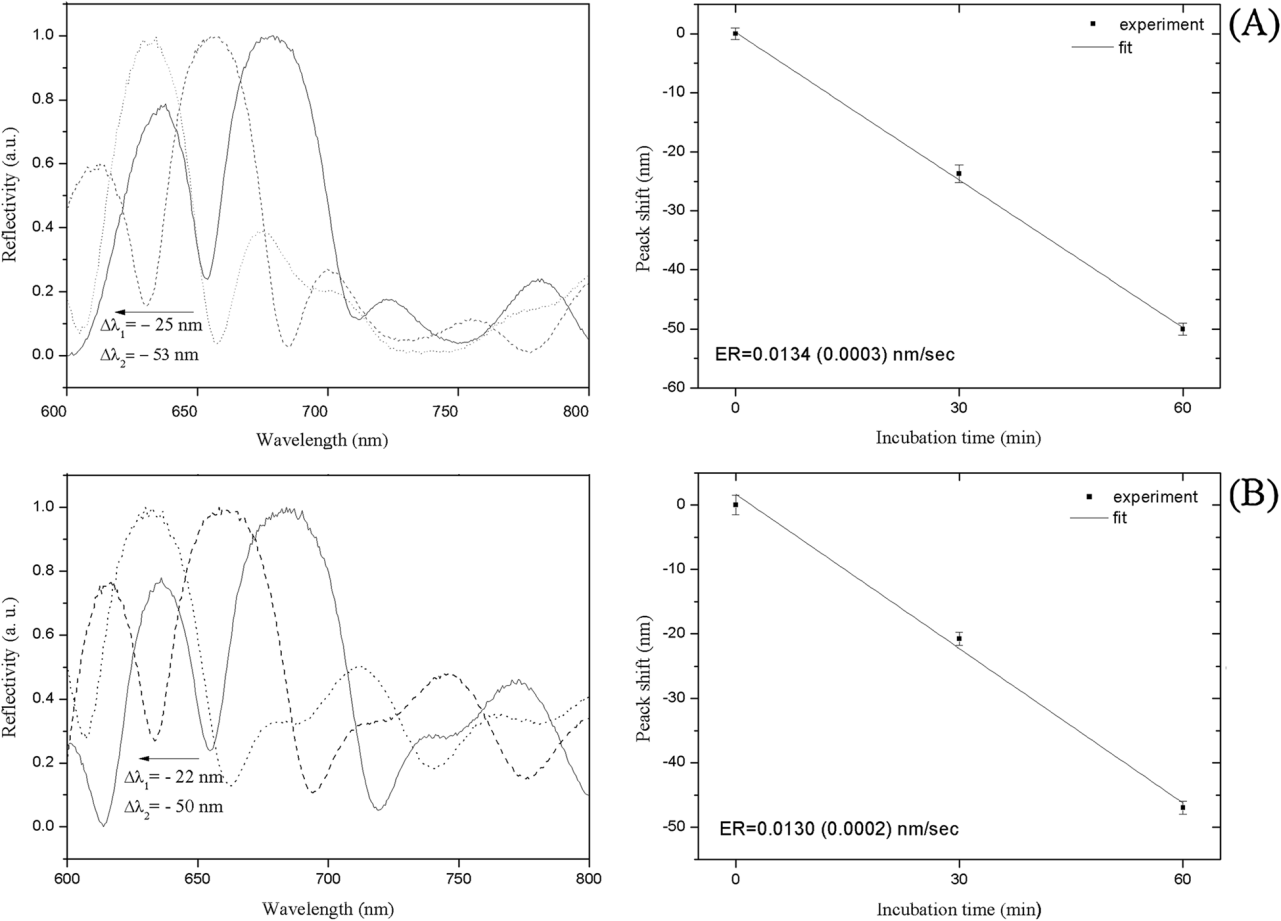
**Reflectivity spectra of APTES- and APDMES**-**modified PSi microcavities before and after incubation in 33% NH**_**3**_**. (A)** Left: reflectivity spectra of APTES-modified PSi microcavity before (solid line) and after 30 (dashed line) and 60 (dotted line) min of incubation in 33% NH_3_ at 55°C. Right: corresponding peak shift vs incubation time. **(B)** Left: reflectivity spectra of APDMES-modified PSi microcavity before (solid line) and after 30 (dashed line) and 60 (dotted line) min of incubation in 33% NH_3_ at 55°C. Right: corresponding peak shift vs incubation time.

The reflectivity spectra and graphs of peak shift vs incubation time for PSi-M_a,b_-NH_2_ microcavities (M_a_ = APTES; M_b_ = APDMES) before and after treatment with 33% aqueous ammonia (17 h, 55°C) used in the standard deprotection condition are reported in Figure 3. The stability of the surfaces was tested by a full dip in ammonia solution for different times. The results showed that the destructive effect of ammonia solution was about the same for both samples: a blue shift of 25 or 50 nm was detected after 30 min or 1 h, respectively, and the complete dissolution of the silicon matrices occurred after 2 h.

**Figure 4:**
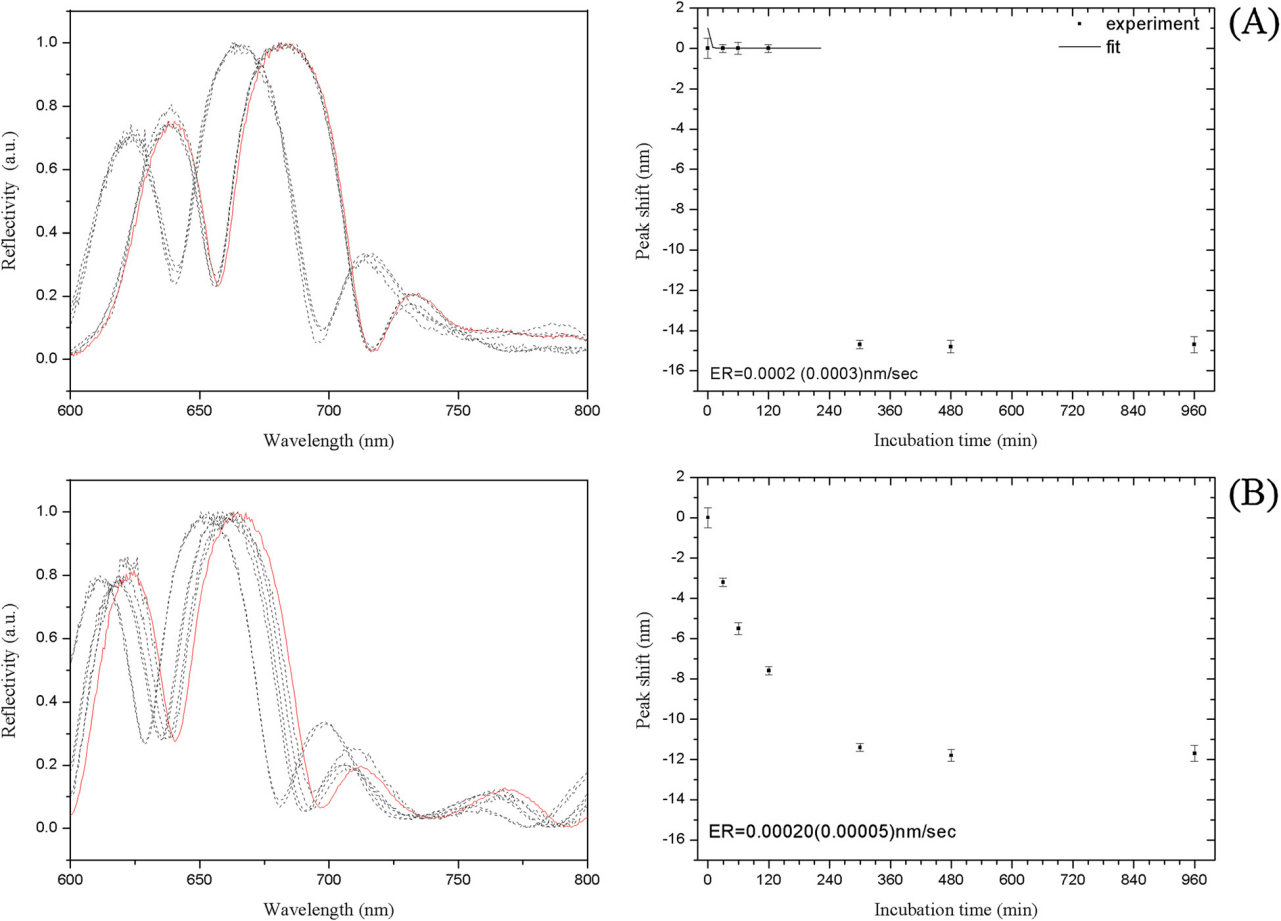
**Reflectivity spectra of APTES- and APDMES-modified PSi microcavities before and after incubation in K**_**2**_**CO**_**3**_**/MeOH dry. (A)** Left: reflectivity spectra of APTES-modified PSi microcavity before (red solid line) and after (dashed line) incubation in K_2_CO_3_/MeOH dry at 55°C for different times. Right: corresponding peak shift vs incubation time. **(B)** Left: reflectivity spectra of APDMES-modified PSi microcavity before (red solid line) and after (dashed line) incubation in K_2_CO_3_/MeOH dry at 55°C for different times. Right: corresponding peak shift vs incubation time.

Because aqueous ammonia could not be used in deprotection steps, we checked the stability of PSi-M_c,d_-NH_2_ (M_c_ = APTES; M_d_ = APDMES) at the so-called ultra-mild deprotection condition (0.05 M K_2_CO_3_/dry methanol at 55°C for 2 h). Sample PSi-M_c_-NH_2_ showed better chemical resistance than sample PSi-M_d_-NH_2_. In particular, a progressive shift of the optical reflectivity spectrum towards shorter wavelength was observed only after more than 2 h of incubation for PSi-M_c_-NH_2_, whereas PSi-M_d_-NH_2_ resulted in being partially stable in ultra-mild deprotection condition only up to 30 min (see plots in Figure 4).

**Figure 5:**
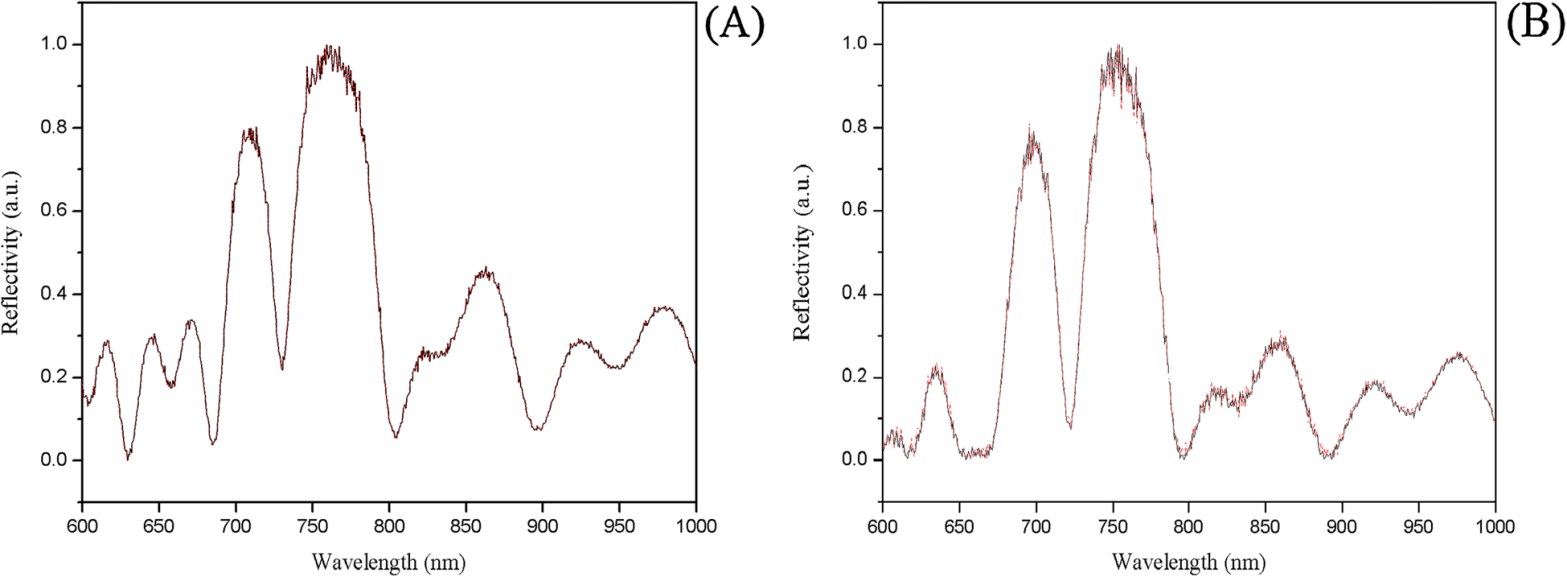
**Reflectivity spectra of APTES- and APDMES-modified PSi microcavities before and after exposure to NH**_**3**_**/MeOH dry and ammonia. (A)** Reflectivity spectra of APTES-modified PSi microcavity before (solid line) and after (red dashed line) exposure to NH_3_/MeOH dry solution at RT. **(B)** Reflectivity spectra of APDMES-modified PSi microcavity before (solid line) and after (red dashed line) exposure to ammonia solution at RT.

As the last route in the deprotection strategy, we tested the saturated dry methanolic ammonia solution. Both the two aminosilane-modified PSi structures (PSi-M_e,f_-NH_2_) were highly stable at this condition. In Figure 5, we have reported the reflectivity spectra of PSi microcavities before and after treatment with NH_3_/MeOH dry. In both cases, any shift cannot be observed, thus confirming the feasibility of this deprotection condition.

**Figure 6:**
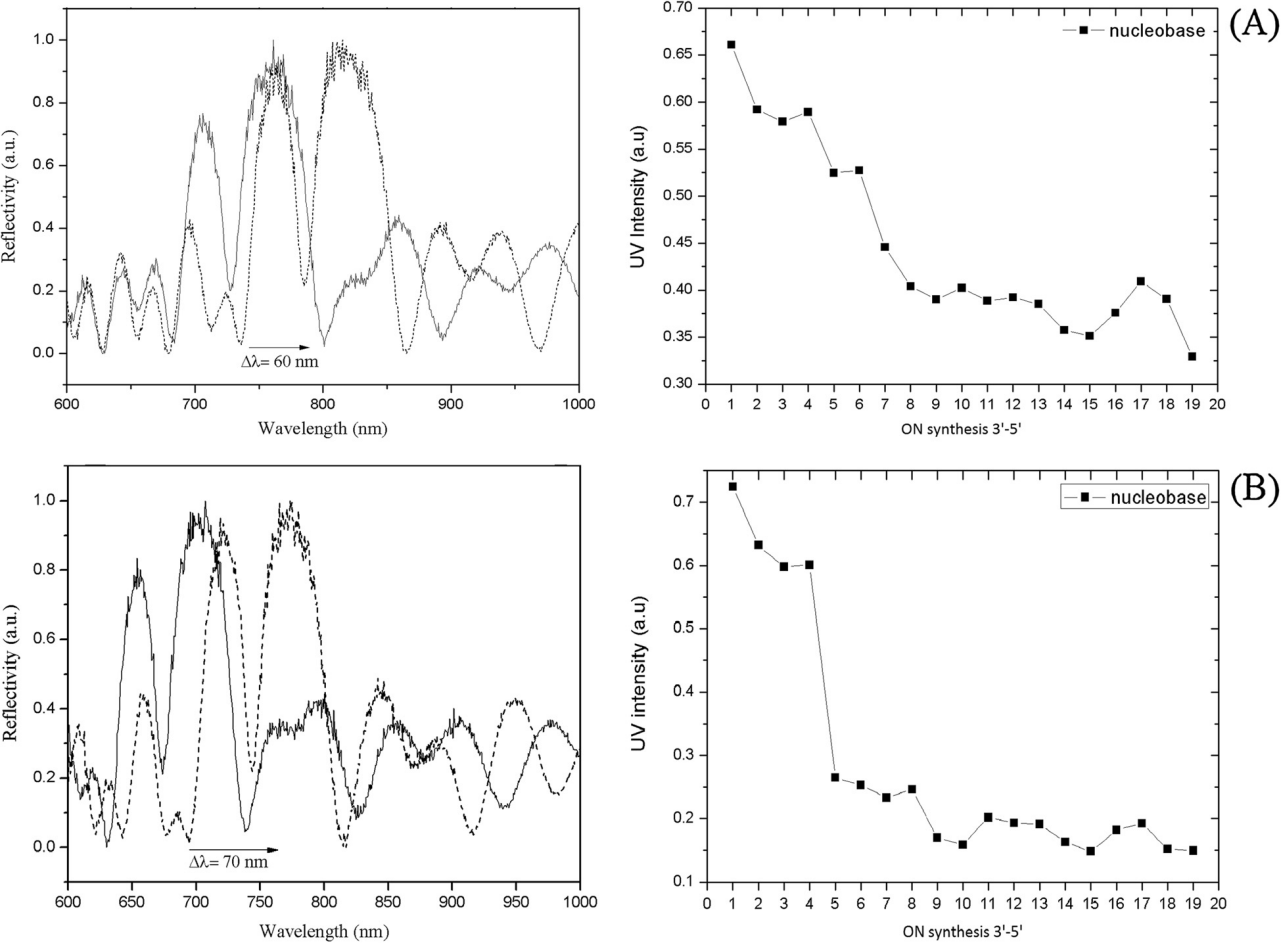
**Reflectivity spectra of APTES- and APDMES-modified PSi microcavities before and after ON synthesis. (A)** Left: reflectivity spectra of APTES-modified PSi microcavity before (solid line) and after (dashed line) ON synthesis. Right: corresponding UV intensity vs ON synthesis. **(B)** Left: reflectivity spectra of APDMES-modified PSi microcavity before (solid line) and after (dashed line) ON synthesis. Right: corresponding UV intensity vs ON synthesis.

Once deprotection conditions were checked and fixed for PSi samples, two microcavities, namely PSi-M_g,h_*-*NH_2_, were used as supports for automated *in situ* solid-phase ON synthesis using the standard phosphoramidite chemistry. The amount of 5′-dimethoxytrityl released after the detritylation step was used to quantify the functionalization yield of each synthesis cycle by UV-vis spectroscopy as shown in Figure 6[16,17]. Up to the fourth coupling cycle, we observed almost the same coupling yield for both aminosilane-functionalized PSi supports. From the fifth cycle on, the coupling yields dropped for both supports, even if higher functionalization yields were generally observed for PSi supports functionalized with APTES.

Figure 6 also shows the reflectivity spectra of devices before and after the *in situ* synthesis process: red shifts of 60 and 70 nm were detected, respectively, for APTES- and APDMES-modified devices, thus indicating that more ON had grown on the latter device with respect to the first one. This experimental result is ascribed to the less steric hindrance of pores due to the thinner APDMES layer, as already demonstrated in our previous work [16].

In both samples, we have measured the red shifts upon exposure to saturated ethanol atmosphere (data not shown here), in order to check if pores could be completely filled up by ON growth: in both cases, we measured red shifts of about 100 nm, just a little bit lower, but of the same order, than those registered in the same experiment after fabrication and silane functionalization. Even if this result is not accurate as standard pore characterization (such as gas adsorption or thermo-porometry), it clearly confirms a minor variation in pore dimensions.

**Figure 7:**
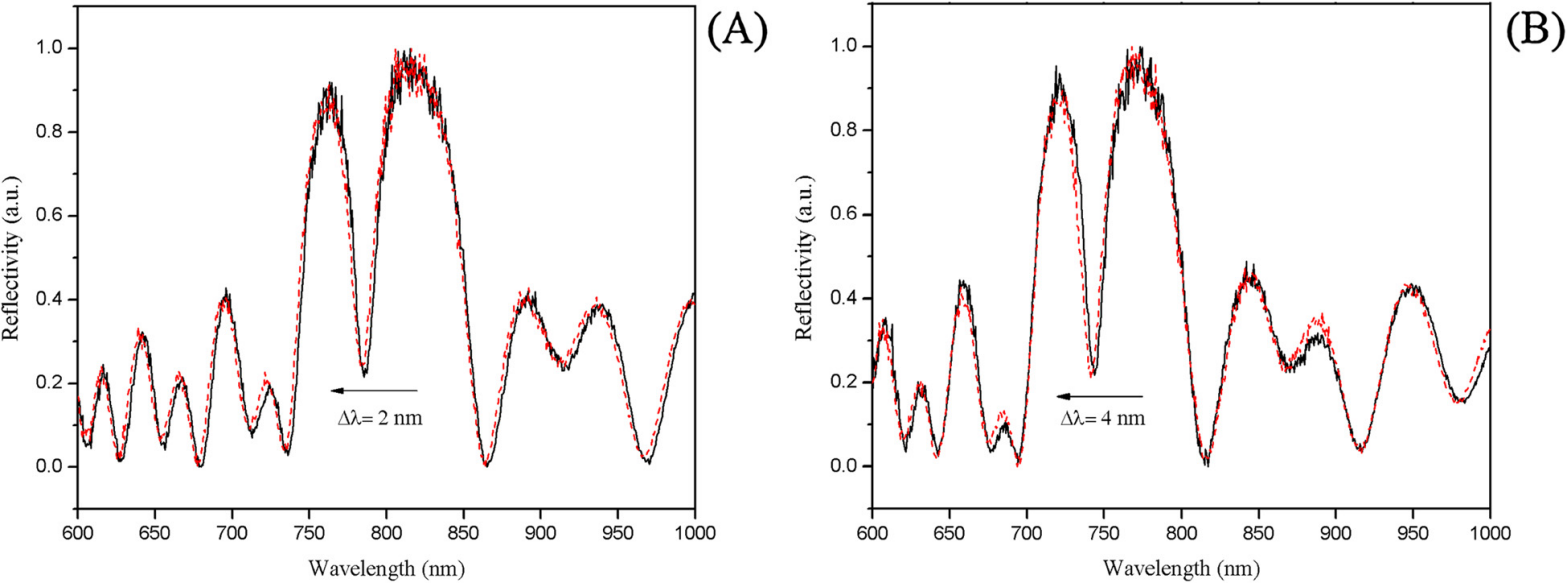
**Reflectivity spectra of APTES- and APDMES-modified PSi microcavities before and after the deprotection process. (A)** Reflectivity spectra of APTES-modified PSi microcavity functionalized with oligonucleotides before (solid line) and after (red dashed line) the deprotection process with gaseous ammonia solution. **(B)** Reflectivity spectra of APDMES-modified PSi microcavity functionalized with oligonucleotides before (solid line) and after (red dashed line) the deprotection process with gaseous ammonia solution.

We demonstrated the ability of NH_3_/dry MeOH solution to completely deprotect the PSi-aminosilane-bound ON by treating the functionalized samples with NH_3_/MeOH at room temperature. We observed by chromatographic analysis that the amide-bound N-2 isobutyryl (on G), N-6 benzoyl (on A) and N-4 benzoyl (on C) were completely cleaved after 3 h at room temperature. Furthermore, it is reported that the ammonia in dry MeOH is able to quickly remove the 2-cyanoethyl phosphate protecting group [15]. This data, together with our findings on the compatibility with the silicon structure, indicates the NH_3_/dry MeOH solution as the best choice to deprotect the exocyclic amino groups of nucleobases and the phosphate groups without promoting the basic hydrolysis on the support, which would instead occur in aqueous conditions. The blue shift of only 2 to 4 nm, which we attribute to the removal of N-2, N-4 and N-6 groups, has been detected after this procedure for *in situ* ON synthesis on PSi-APTES or PSi-APDMES supports, respectively (see plots in Figure 7).

## Conclusions

In the present study, we propose and validate by optical measurements a new method to achieve the *in situ* synthesis of tailored oligonucleotide sequences on porous silicon supports suitable for label-free optical biosensing. In particular, we demonstrate that, differently from aqueous ammonia, the use of dry ammonia in methanol allows the effective deprotection of nucleobases without harming the structural integrity of the porous silicon matrix, thus opening the way for the direct growing of mixed-sequence ONs on optically active PSi supports using exclusively inexpensive standard phosphoramidites. A 19-mer mixed-sequence 5′-GATTGATGTGGTTGATTTT-3′ has been synthesized in mesoporous PSi microcavities, resulting in a medium-yield process, mainly due to the average pore size (about 20 nm). PSi photonic devices with pore dimensions greater than that value, but always compatible with high optical quality response in the visible-near-infrared, therefore between 50 and 100 nm, will be considered in the next experiments, in order to maximize yield synthesis. Moreover, more stable PSi supports could also be considered, such as those produced by thermal acetylation, which maintains pore size and makes it very stable from the chemical point of view [18].

## Competing interests

The authors declare that they have no competing interests.

## Authors’ contributions

MT performed the experiments. LDS and IR designed the research. MT and IR analysed the data and wrote the paper. LDS and NB corrected the paper. MT prepared and characterized the samples. GO, SDE and FN performed the oligonucleotide synthesis and characterization. IvR and GP have given final approval of the version to be published. All authors read and approved the final manuscript.
